# Sudden infant death syndrome prevention

**DOI:** 10.1186/s12887-021-02536-z

**Published:** 2021-09-08

**Authors:** Sophie Jullien

**Affiliations:** Barcelona Institute for Global Health, University of Barcelona, Barcelona, Spain

**Keywords:** Prevention, Sudden infant death syndrome, Sleeping practices, Infant

## Abstract

We looked at existing recommendations and supporting evidence for successful strategies to prevent the sudden infant death syndrome (SIDS).

We conducted a literature search up to the 14th of December 2020 by using key terms and manual search in selected sources. We summarized the recommendations and the strength of the recommendation when and as reported by the authors. We summarized the main findings of systematic reviews with the certainty of the evidence as reported.

Current evidence supports statistical associations between risk factors and SIDS, but there is globally limited evidence by controlled studies assessing the effect of the social promotion strategies to prevent SIDS through knowledge, attitude and practices, due to obvious ethical reasons. A dramatic decline in SIDS incidence has been observed in many countries after the introduction of “Back to Sleep” campaigns for prevention of SIDS. All infants should be placed to sleep in a safe environment including supine position, a firm surface, no soft objects and loose bedding, no head covering, no overheating, and room-sharing without bed-sharing. Breastfeeding on demand and the use of pacifier during sleep time protect against SIDS and should be recommended. Parents should be advised against the use of tobacco, alcohol and illicit drugs during gestation and after birth.

## Background

### Introduction

The World Health Organization (WHO) European Region is developing a new pocket book for primary health care for children and adolescents in Europe. This article is part of a series of reviews, which aim to summarize the existing recommendations and the most recent evidence on preventive interventions applied to children under 5 years of age to inform the WHO editorial group to make recommendations for health promotion in primary health care. In this article, we looked at existing recommendations and supporting evidence for successful strategies to prevent the sudden infant death syndrome (SIDS).

### What is the sudden infant death syndrome?

SIDS is ‘the sudden death on an infant under one year of age which remains unexplained after a thorough case investigation, including performance of a complete autopsy, examination of the death scene, and review of the clinical history’ [1]. The sudden unexpected infant death (SUID) or sudden unexpected death in infancy is a broader term referring to ‘a sudden and unexpected death, whether explained or unexplained, occurring during infancy’ and includes the SIDS and other sleep-related infant death such as ill-defined death and accidental suffocation and strangulation in bed [2]. Therefore, for any SUID, when the cause of death after case investigation is not attributed to any explained cause such as suffocation, asphyxia, infection or metabolic diseases, the case is classified as SIDS, which is an ultimate diagnosis reached by exclusion.

### Context

Although defined by an unexplained origin, several risk factors have been associated with the incidence of SIDS. Despite the success of several preventive campaigns started in the 1990’s targeting modifiable risk factors related with the SIDS, it remains a leading cause of infant mortality in high-income countries. The rate of SIDS was estimated at 19.8 per 100,000 live births among 14 European countries between 2005 and 2015, ranging from 1.4 to 29.2 between countries [3]. It is therefore imperative to identify and assess the effective strategies to prevent SIDS.

## Key questions

1. Which are the most important risk factors associated with the SIDS?
2. Which are the successful strategies to prevent SIDS?

## Search methods and selected manuscripts

We described the search methods, data collection and data synthesis in the second paper of this supplement (Jullien S, Huss G, Weige R. Supporting recommendations for childhood preventive interventions for primary health care: elaboration of evidence synthesis and lessons learnt. BMC Pediatr. 2021. 10.1186/s12887-021-02638-8).

We conducted the search up to the 14th of December 2020, by manual search and by using the search terms “sudden death”, “unexpected death”, “sudden infant death syndrome”, and “SIDS”. We found a bulletin from the WHO with a short comment on the topic. No document was identified from the US Preventive Services Task Force (USPSTF) website, but we found their position published through the American Academy of Pediatrics (AAP), in a manuscript that was first published in 2011, with updated recommendations in 2016 [2]. The recommendations from the PrevInfad workgroup (Spanish Association of Primary Care Pediatrics) were also published in 2016, together with their supportive document [4]. The Centers of Disease Control and Prevention (CDC) supports the AAP recommendations and summarize them in their website [5, 6]. We found 72, 36, 18, and 10 documents by using the search terms cited above, respectively, in the National Institute for Health and Care Excellence (NICE) official website. Out of them, we retrieved two NICE guidelines that addressed SIDS, but recommendations were from a single guideline [7]. The search in the Cochrane library returned 17 reviews and no protocols. By screening the titles and abstracts, we included one systematic review [8].

All the included manuscripts for revision in this article are displayed in Table 1.

**Table 1:** Included manuscripts for revision

Sources	Final selected manuscripts
**WHO**	WHO bulletin with reference to the 2011 recommendations from the AAP [9]
**USPSTF**	No document identified in their website, but recommendations published through the AAP (see below)
**PrevInfad**	Recommendations and supporting document [4]
**CDC**	Promotion of the recommendations from the AAP [5, 6]
**NICE**	‘Postnatal care up to 8 weeks after birth’ guidelines [7]
**AAP**	Updated 2016 recommendations [2] Evidence base document for 2016 recommendations [10]
**Cochrane Library**	Psaila 2017 (infant pacifier) [8]

*Abbreviations*: *AAP* American Academy of Pediatrics, *CDC* Centers for Disease Control and Prevention, *NICE* National Institute for Health and Care Excellence, *PrevInfad* PrevInfad workgroup from the Spanish Association of Primary Care Pediatrics, *USPSTF* US Preventive Services Task Force, *WHO* World Health Organization

## Existing recommendations

Both the WHO and CDC promote the AAP recommendations. In the NICE guideline ‘Postnatal care up to 8 weeks after birth’ the recommendations provided are as follows [7]:

- “Recognise that co-sleeping can be intentional or unintentional. Discuss this with parents and carers and inform them that there is an association between co- sleeping (parents or carers sleeping on a bed or sofa or chair with an infant) and SIDS.”
- “Inform parents and carers that the association between co-sleeping (sleeping on a bed or sofa or chair with an infant) and SIDS is likely to be greater when they, or their partner, smoke.”
- “Inform parents and carers that the association between co-sleeping (sleeping on a bed or sofa or chair with an infant) and SIDS may be greater with parental or carer recent alcohol consumption, or parental or carer drug use, or low birthweight or premature infants.”

The AAP and the PrevInfad documents, published the same year, provide a list of very similar recommendations that we summarized together with the strength of each recommendation (as per their authors) in Table 2. Many of the modifiable and non-modifiable risk factors identified for SIDS are very similar to those for other sleep-related infant deaths such as suffocation or asphyxia. In their document, the AAP provides recommendations for a safe sleep environment with the aim of reducing all sleep-related infant deaths [2]. Recommendations related to sleeping environment apply to infants up to 12 months of age.

**Table 2:** Summary of recommendations and strength of recommendations by PrevInfad and the AAP

Effective strategies to reduce SIDS	PrevInfad 2016^**a**^	AAP 2016^**a**^
**Modifying behaviours and care related to the sleeping environment and nutrition**		
Supine position for sleeping	Grade A	Grade A
Supervised awake tummy time	Recommended but not graded	Grade B
Firm surface for sleeping	Recommended but not graded	Grade A
Soft objects and loose bedding away from the sleep area	Recommended but not graded	Grade A
Avoid overheating and head covering	Grade I	Grade A
Room-sharing with the infant on a separate sleep surface	Grade B	Grade A
No bed-sharing if father or mother are tobacco smokers, have consumed alcohol, anxiolytic, antidepressant or hypnotic drugs and in case of extreme exhaustion.	Grade B	Recommended but not graded
No routine use of commercial devices that are inconsistent with safe sleep recommendations.	Not reported	Grade B
Consider offering a pacifier at naptime and bedtime	Grade B	Grade A
Breastfeeding on demand	Grade A	Grade A
**Counselling to modify behaviours and care related to maternal factors**		
Regular prenatal care	Grade B	Grade A
Avoid smoke exposure during pregnancy and after birth	Grade A	Grade A
Avoid alcohol and illicit drug use during pregnancy and after birth	Grade B	Grade A
**Infant-related factors**		
Prematurity and low birth weight	Not graded	Not reported
Sibling with SIDS	Not graded	Not reported
**Other strategies to modify behaviours**		
Infants should receive immunizations following respective national immunization programme	Grade I	Grade A
No use of cardiorespiratory monitors at home as a strategy to reduce the risk of SIDS	Grade I	Grade A
Health care providers, staff in newborn nurseries and neonatal intensive care units, and childcare providers should endorse and model the SIDS risk-reduction recommendations from birth.	Not reported	Grade A
Media and manufacturers should follow safe sleep guidelines in their messaging and advertising.	Not reported	Grade A
Continue the “Safe to Sleep” campaign, focusing on ways to reduce the risk of all sleep-related infant deaths, including SIDS, suffocation, and other unintentional deaths. Paediatricians and other primary care providers should actively participate in this campaign.	Not reported	Grade A
Continue research and surveillance on the risk factors, causes, and pathophysiologic mechanisms of SIDS and other sleep-related infant deaths, with the ultimate goal of eliminating these deaths entirely.	Not reported	Grade C
There is no evidence to recommend swaddling as a strategy to reduce the risk of SIDS.	Not reported	Grade C

*Abbreviations*: *AAP* American Academy of Pediatrics, *PrevInfad* PrevInfad workgroup from the Spanish Association of Primary Care Pediatrics; SIDS: sudden infant death syndrome

^a^The definitions of the five grades to describe the strength of the recommendations are reported in (Jullien S, Huss G, Weige R. Supporting recommendations for childhood preventive interventions for primary health care: elaboration of evidence synthesis and lessons learnt. BMC Pediatr. 2021. 10.1186/s12887-021-02638-8)

## Existing evidence

With the aim to analyse preventive measures to reduce SIDS, factors that increase or decrease the risk of SIDS have been identified. However, the identification of statistical associations between risk factors and SIDS does not prove a causal link or mechanistic explanation. The different institutions developed their recommendations based on these statistical associations together with the assessment of other factors such as the balance between potential benefit from reducing the risk and any harm derived from the preventive strategy. Although the evidence exposed below show the association between identified risk factors and SIDS, there is limited evidence assessing the effect of the recommendations through knowledge, attitude, and practices, with the exception of the sleep position [4, 11].

We mainly retrieved the evidence from the two supportive documents developed for the PrevInfad and AAP recommendations [2, 4, 10]. Therefore, the references cited below were used in these documents and do not correspond with an additional literature review conducted by the authors of this summary document. As already indicated by the AAP, there are no randomized controlled trials (RCTs) with regard to SIDS and other sleep-related deaths. Evidence mainly derives from case-control studies and national pre and post intervention data. Currently, it is very unlikely that any clinical trial will be initiated to assess effectiveness of known risk factors due to obvious ethical reasons. The only Cochrane review identified aimed to assess the use of infant pacifiers for reduction of SIDS but no RCT addressing the topic was found. We summarize below the evidence supporting the recommendations addressing the most relevant or controversial risk factors.

### Modifying behaviours and care related to the sleeping environment and nutrition

#### Supine position for sleeping

##### Recommendations

“Avoid prone sleeping position in infants less than 6 months old. Sleeping in supine decubitus position is the safest and clearly preferable to lateral decubitus. Only in a specific medical indication (severe gastroesophageal reflux, active respiratory illness in preterm infants and certain upper way malformations) can prone decubitus be recommended.” (*Grade A recommendation*) [4].

“To reduce the risk of SIDS, infants should be placed for sleep in the supine position (wholly on the back) for every sleep period by every caregiver until 1 year of age. Side sleeping is not safe and is not advised.” (*Grade A recommendation*) [2].

##### Evidence

This is the main modifiable risk factor identified for SIDS. Consistent findings across the world and decreasing trend on the incidence of SIDS in countries that have implemented the ‘Back to Sleep’ recommendations support the hypothesis that the supine position for sleep protects against SIDS [4]. Indeed, case-control studies, conducted in Europe and the United States indicate that the prone position during sleep increases the risk of SIDS as compared to supine position with adjusted odds ratio (AOR) ranging from 2.3 and 13.1 [12–16]. Similarly, the lateral side has been associated with increased risk of SIDS when compared to supine position, with AOR ranging from 1.31 to 2 [13–15]. These five case-control studies were conducted in the US [12, 13, 16], the UK [14] and in 20 regions of Europe [15] from 1992 and 2000, including 1432 SIDS cases and 3905 matched controls. In addition, countries with preventive campaigns for avoiding prone position in infants during sleep that have been successful for reducing the prevalence of infants sleeping in such position have estimated a 30 to 50% decrease in the mortality associated to SIDS [4].

Supine position does not increase the risk of choking and aspiration [2, 4]. Only infants with certain upper airway disorders such as type 3 or 4 laryngeal clefts in which the risk of death from gastroesophageal reflux disease may outweigh the risk of SIDS can be considered to be placed in prone position during sleep [2].

#### Supervised awake tummy time

##### Recommendations

“When awake, infants can be placed in prone position with supervision.” (Recommended but not graded) [4].

“Supervised, awake tummy time is recommended to facilitate development and to minimize development of positional plagiocephaly.” (*Grade B recommendation*) [2].

##### Evidence

Sustained supine position combined with restricted motor abilities lead to postural plagiocephaly [4]. In addition, prone position facilitates the development of the upper shoulder girdle strength [2]. Therefore, although there is no data to support this recommendation and to establish the frequency and duration of it, experts recommend “a certain amount of prone positioning, or ‘tummy time,’ while the infant is awake and being observed” [17].

#### Firm surface for sleeping

##### Recommendations

“Firm surfaces should always be used: the mattresses must be firm and maintain their shape even when covered with the sheets, so that there are no gaps left between the mattress and the crib railing. Adjustable sheets and specific bedding should be used.” [4].

“Infants should be placed on a firm sleep surface (eg, mattress in a safety-approved crib) covered by a fitted sheet with no other bedding or soft objects to reduce the risk of SIDS and suffocation.” (*Grade A recommendation*) [2].

##### Evidence

Soft sleep surface has consistently been reported as a risk factor for SIDS. A case-control study conducted in the US among 260 SIDS cases and 260 matched living controls, showed an association between soft sleep surface and a higher risk of SIDS (AOR 5.1 [95% CI: 2.9 to 9.2]) [12]. The risk was significantly higher when prone position and soft sleep surface were combined (AOR 21.0 [95% CI: 7.8 to 56.2]) [12]. Soft mattresses could create a pocket around the infant within which the CO2 dispersal is limited, increasing the risk of rebreathing or suffocation in infants placed in prone position [2, 18].

#### Soft objects and loose bedding away from the sleep area

##### Recommendations

“Other loose accessories such as blankets, quilts and pillows, cushions, soft objects and neck pendants” should be kept away from the infant’s sleep area [4].

“Keep soft objects and loose bedding away from the infant’s sleep area to reduce the risk of SIDS, suffocation, entrapment, and strangulation.” [2].

##### Evidence

Several publications pointed out that soft objects (pillows, pillow-like toys, quilts, comforters, sheepskins) and loose bedding (blankets, nonfitted sheets) can cause the obstruction of an infant’s external airways, leading to an increased risk of suffocation, rebreathing, and SIDS [2, 10]. In an already mentioned study, the use of pillow and covering the head or face with bedding were associated to an increased risk of SIDS (AOR 3.1 [95% CI 1.6 to 5.8] and AOR 2.5 [95% CI 1.2 to 5.2]) [12]. A higher risk was found when the use of pillow was combined with prone position (AOR 11.8 [95% CI 4.0 to 34.4]) [12]. In another study conducted in the US among 206 SIDS cases showed that the use of comforters (AOR 2.46) and pillows (AOR 3.31) increased the risk of death (95% CI not provided, but *p* ≤ 0.05 for both comparisons) [19]. Other studies reported that infants victim of SIDS were found in supine position with their head covered by loose bedding.

#### Avoid overheating and head covering

##### Recommendations

“Avoid overheating and avoid the head to be covered while sleeping” “The recommendation to prevent the head from covering is to put the infant at the foot of the bed and the blanket up to the chest.” (*Grade I recommendation*) [4].

“Avoid overheating and head covering in infants.” “In general, infants should be dressed appropriately for the environment, with no greater than 1 layer more than an adult would wear to be comfortable in that environment.” (*Grade A recommendation*) [2].

##### Evidence

Overheating has been identified as a risk factor for SIDS, especially when the head is covered. Both the AAP and PrevInfad have stated that several studies had shown that overheating (including external temperature and the child’s clothes) was associated with an increased risk of SIDS, but that it was difficult to provide any specific room temperature recommendation as the definition of overheating varies across studies [2, 4]. When looking at the ‘several studies’ mentioned above, we found no references from PrevInfad, and four references cited in the AAP document. Three manuscripts are case-control studies published between 1990 and 2002 that showed an increased risk of SIDS when infants were heavily wrapped, when the heating was on all night, or when the infants slept with two or more layers of clothing, showing a small effect or a broad confidence interval [20–22]. The fourth study analysed data from one of the three cited case control by the same first author, and a prospective cohort, to emphasize the increased risk of SIDS when the prone position is associated with other risk factors including overheating [23]. To avoid overheat, several strategies have been put in place. PrevInfad recommends a temperature of 20 to 22 °C and to avoid excessive clothing, especially if the infant has fever. AAP recommends that ‘in general, infants should be dressed appropriately for the environment, with no greater than one layer more than an adult would wear to be comfortable in that environment’ and that ‘parents and caregivers should evaluate the infant for signs of overheating, such as sweating or the infant’s chest feeling hot to the touch’. Both identities agree that ‘there is currently insufficient evidence to recommend the use of a fan as a SIDS risk-reduction strategy’.

A systematic review including 10 case-control studies conducted between 1958 and 2003 found that the prevalence of head covering was higher in SIDS cases (24.6% [95% CI 22.3 to 27.1%]) than in controls (3.2% [95% CI 2.7 to 3.8%]) [24]. The AOR was 16.9 (95% CI 12.6 to 22.7) and the risk associated to SIDS was consistently significant across studies. The review did not establish a causal mechanism between head covering and SIDS, but the authors concluded that head covering is a major modifiable risk factor associated with SIDS. With a potential high attributable risk of 27.1% and the ease of adopting this measure with low cost and no adverse effect, avoiding head covering was adopted as a recommendation to decrease deaths related to SIDS [25]. As a strategy to avoid head covering, a ‘Feet to foot’ campaign was initiated, which recommends placing the baby at the foot of the cot. However, this strategy was established following common sense, but there is no evidence showing that this measure does reduce head covering and has any impact on SIDS.

Overall, it seems that there is low quality evidence regarding overheating and head covering and that current strategies are based on common sense that have not been proved to reduce SIDS.

#### Room-sharing with the infant on a separate sleep surface

##### Recommendations

“The crib in the parents’ bedroom is the safest place.” (*Grade B recommendation*) [4].

“Recommend against co-sleeping if father or mother are tobacco smokers, have drunk alcohol, anxiolytic, antidepressant or hypnotic drugs have been used and in case of extreme exhaustion. Co-sleeping is advised against also in sofas, armchairs or any other place but the bed.” (*Grade B recommendation*) [4].

“Inform parents that there is not enough evidence to recommend against bed-sharing when infants are breastfed and there are no other risk factors” (*Grade I recommendation*) [4].

“It is recommended that infants sleep in the parents’ room, close to the parents’ bed, but on a separate surface designed for infants, ideally for the first year of life, but at least for the first 6 months.” (*Grade A recommendation*) [2].

“Infants should never be placed on a couch or armchair for sleep.” [2].

##### Evidence

Co-sleeping and bed-sharing do not mean the same. The term co-sleeping refers to parents and infant sleeping in close proximity, which can be bed-sharing (sleeping on the same surface) or sleeping in the same room in close proximity on separate surfaces [10]. Room-sharing has been shown to reduce the risk of SIDS by as much as 50% [2, 4]. However, bed-sharing between parents and infant remains highly controversial. While bed-sharing has been associated with an increased risk of SIDS, bed-sharing has also been assessed to improve attachment and breastfeeding, considered as a protecting factor to SIDS (see below).

A meta-analysis published in 2012 and including 11 studies conducted between 1987 and 2006 looked at the association between bed-sharing and SIDS. Authors found an increased risk of SIDS among those bed-sharing with an odds ratio (OR) of 2.89 (95% CI: 1.99 to 4.18) and an increased risk among smoking mothers (OR 6.27 [95% CI 3.94 to 9.99]; 4 studies) [26]. Carpenter et al. pooled data from five case-control studies including Scotland, Germany, Ireland, other European countries, and New Zealand to look at the same association of bed-sharing and the risk of SIDS, among breastfed infants with non-smoking parents and with no maternal use of alcohol or drugs, with no other associated risk factors [27]. They found an increased risk of SIDS among infants with bed-sharing versus room sharing with an AOR of 2.7 (95% CI 1.4 to 5.3) and a higher risk in infants less than 3 months (AOR 5.1 [95% CI 2.3 to 11.4]).

Blair et al. had opposite findings when assessing the same association of bed sharing with SIDS among infants without other risk factors from two different case-control studies conducted between 1993 and 2006 in the UK [28]. They found no association between bed sharing and SIDS globally (OR 1.1 [IC 95% 0.6 to 2]) and among infants under 3 months of age (OR 1.6 [95% CI 0.96 to 2.7]). Among infants above 3 months of age, authors found bed sharing to be protector for SIDS, with an OR of 0.1 (95% CI 0.01 to 0.5). These findings were independent of whether the infant was breastfed or not. When looking at this association in presence of parents who consumed tobacco or alcohol, they found similar findings to Carpenter.

Facing these contradicting findings and recommendations between Carpenter et al. and Blair et al., the US task force requested an independent review of both manuscripts, reported by the AAP. They concluded that both studies have strengths and weaknesses, and that both studies lacked power to examine the association in subgroups of children (under or above 3 months of age). “Clearly, these data do not support a definitive conclusion that bed-sharing in the youngest age group is safe, even under less hazardous circumstances.” [10].

In summary, there is a lack of evidence to determine the balance between harm and benefits of bed-sharing among infants without other risk factors associated (parental use of tobacco or alcohol), taking breastfeeding into consideration. Accordingly, in case of breastfed infants with no other risk factors, PrevInfad recommends to inform parents that there is not enough evidence to recommend against bed-sharing (*Grade I recommendation*) [4]. However, there are specific circumstances that have been shown to substantially increase the risk of SIDS, independently to the form of feeding and that should be avoided. Those are summarized by the AAP as follows, and are in agreement with the PrevInfad and NICE recommendations [4, 7, 10]: “when one or both parents are smokers, even if they are not smoking in bed (OR 2.3 to 21.6); when the mother smoked during pregnancy; when the infant is younger than four months of age, regardless of parental smoking status (OR 4.7 to 10.4); when the infant is born preterm and/or with low birth weight; when the infant is bed-sharing on excessively soft or small surfaces, such as waterbeds, sofas and armchairs (OR 5.1 to 66.9); when soft bedding accessories such as pillows or blankets are used (OR 2.8 to 4.1); when there are multiple bed sharers (OR 5.4); when the parent has consumed alcohol (OR 1.66 to 89.7) and/or illicit or sedating drugs; and when the infant is bed-sharing with someone who is not a parent (OR.5.4).”

#### Consider offering a pacifier at naptime and bedtime

##### Recommendations

“Not rejecting the use of a pacifier during sleeping time in the first year of life seems to be a cautious measure.” (*Grade B recommendation*) [4].

“Consider offering a pacifier at naptime and bedtime” (*Grade A recommendation*) [2].

“Offer a pacifier to the infant when put to sleep in supine position, and do not reinsert it once the infant is asleep. If the infant refuses the pacifier, do not force him or her to use it.” [2, 4].

“For breastfed infants, pacifier introduction should be delayed until breastfeeding is firmly established” [2] or until the infant is 1 month of age [4].

##### Evidence

Although the mechanism is unclear, the use of pacifier during the sleep has a protective effect on SIDS [2, 4]. A Cochrane review was published in 2017, after the development of both the PrevInfad and the AAP recommendations [8]. The aim of this review was to evaluate the use of infant pacifiers versus no pacifiers during sleep in reducing the risk of SIDS. However, the review authors found no randomized controlled trials addressing this topic.

Recommendations are mainly based on findings from another systematic review that was conducted by Hauck et al. and included case control studies published between 1993 and 2004 [29]. A protector effect of pacifier was shown for usual pacifier use (AOR 0.71 [95% CI 0.59 to 0.85]; 4 studies) and for use of pacifier in the last sleep (AOR 0.39 [95% CI 0.31 to 0.50]; 7 studies). Authors also estimated the number needed to treat as 2733 (95% CI 2416 to 3334), meaning that one SIDS death could be prevented for every 2733 infants using a pacifier during the sleep.

Pacifier can be introduced as soon as desired after birth in not breastfed infants, but it is recommended to delay its introduction in breastfed infants until breastfeeding is well established [2, 4]. There is however a lack of evidence to confirm the belief that the use of pacifier interferes with breastfeeding [4].

#### Breastfeeding on demand

##### Recommendations

“Recommend breast-feeding on demand.” (*Grade A recommendation*) [4].

“Unless contraindicated, mothers should breastfeed exclusively or feed with expressed milk (i.e., not offer any formula or other nonhuman milk- based supplements) for 6 months, in alignment with recommendations of the AAP” (*Grade A recommendation*) [2].

##### Evidence

Breastfeeding is a clear protective factor for SIDS. Exclusive breastfeeding is recommended for the first 6 months of life, in line with global recommendations [30]. A systematic review included 18 case control studies (published between 1976 and 2009) for meta-analysis [31]. The univariate analysis showed a protector effect of any breastfeeding (any amount for any duration) versus no breastfeeding (OR 0.40 [95% CI 0.35 to 0.44]; 18 studies), which was maintained with multivariate analysis from seven of the included studies (AOR 0.55 [95% CI 0.44 to 0.69]; 7 studies). The protective effect was higher in infants who were exclusively breastfed for any duration in univariate analysis (OR 0.27 [95% CI 0.24 to 0.31]; 8 studies), with no data provided in the included studies allowing multivariate analysis [31].

### Counselling to modify beneficial behaviours and care related to maternal factors

#### Regular prenatal care

##### Recommendations

“Recommend appropriated control of pregnancy and perinatal period.” (*Grade B recommendation*) [4].

“Pregnant women should obtain regular prenatal care” (*Grade A recommendation*) [2].

##### Evidence

This recommendation is mainly based on the findings of a case control study nested in a large cohort of all live births in the US between 1995 and 1998, which aimed to identify maternal and obstetric risk factors for SIDS [32]. From 12,404 cases (SIDS) and 49,616 controls, authors found an increased risk for SIDS when there was no prenatal care (OR 1.70 [95% CI 1.44 to 2.0]).

#### Avoid smoke exposure during pregnancy and after birth

##### Recommendations

“Recommend against tobacco smoking to parents, especially to the mother during pregnancy, although also after delivery. Don’t allow anybody smoking in the infants’ presence.” (*Grade A recommendation*) [4].

“Smoking during pregnancy, in the pregnant woman’s environment, and in the infant’s environment should be avoided.” (*Grade A recommendation*) [2].

##### Evidence

Maternal smoking is an independent risk factor for SIDS. This association has been found independently for both maternal smoking during pregnancy and after birth, from several studies [2, 4]. The large case-control nested study mentioned above for prenatal care, also associated maternal smoking during pregnancy with an increased risk of SIDS (OR 3.19 [95% CI 3.03 to 3.37]) [32]. Several studies have confirmed the association between foetal nicotine exposure and neuropathological and neurochemical anomalies. These anomalies are translated into dysregulation of the autonomic nervous system, prompting disruption of ventilation and cardiac rhythm control, leading to sudden and unexpected death [33]. In addition, it is also well known that smoke exposure is associated with an increased risk of preterm birth and low birth weight, which are both identified risks for SIDS [2].

Regarding exposure to smoke in any circumstances such as in the same house or car, 13 studies found that the maternal or paternal habit of smoking after birth increased the risk of SIDS 2.31 times (95% CI 2.02 to 2.59%) [4]. The association between smoke exposure and SIDS is dose dependent. The risk increases substantially when there is bed sharing between the infant and the smoker, even if the adult does not smoke in bed [10].

#### Avoid alcohol and illicit drug use during pregnancy and after birth

##### Recommendations

“Avoid the prenatal and postnatal use of alcohol and illegal drugs.” (*Grade B recommendation*) [4].

“Avoid alcohol and illicit drug use during pregnancy and after the infant’s birth.” (*Grade A recommendation*) [2].

##### Evidence

The use of alcohol or illicit drugs during prenatal (periconceptional and gestational) and postnatal periods has been associated with increased risk of SIDS [2, 4]. Similarly to smokers, the risk increases when alcohol or drug user share the bed with the infant [2, 4].

## Summary of findings

- Current evidence supports statistical associations between risk factors and SIDS, but there is globally limited evidence by controlled studies assessing the effect of the social promotion strategies to prevent SIDS through knowledge, attitude and practices, due to obvious ethical reasons.
- A dramatic decline in SIDS incidence has been observed in many countries after the introduction of “Back to Sleep” campaigns for prevention of SIDS.
- All infants should be placed to sleep in a safe environment including supine position, a firm surface, no soft objects and loose bedding, no head covering, no overheating, and room-sharing without bed-sharing.
- Breastfeeding on demand and the use of pacifier during sleep time protect against SIDS and should be recommended.
- Parents should be advised against the use of tobacco, alcohol and illicit drugs during gestation and after birth.
- The American Academy of Pediatrics recommendations updated in 2016 are the most comprehensive resume about SIDS prevention.

## Data Availability

Not applicable.
